# Focused cardiac ultrasound: a training course for pediatric intensivists and emergency physicians

**DOI:** 10.1186/1472-6920-14-25

**Published:** 2014-02-05

**Authors:** Heloisa A Gaspar, Samira S Morhy, Alessandro C Lianza, Werther B de Carvalho, Jose L Andrade, Rogério R do Prado, Cláudio Schvartsman, Artur F Delgado

**Affiliations:** 1 Pediatric Intensive Care - Instituto da Criança do Hospital das Clínicas da Faculdade de Medicina, São Paulo University, Rua do Carreiro de Pedra 111 apto 152C, Jd. Caravelas, CEP 04728-020 São Paulo, Brazil; 2 Radiology Department - Instituto da Criança do Hospital das Clínicas da Faculdade de Medicina, São Paulo University, São Paulo, Brazil; 3 Department of Statistics, Instituto da Criança do Hospital das Clínicas da Faculdade de Medicina, São Paulo University, São Paulo, Brazil; 4 Emergency Medicine Department, Instituto da Criança do Hospital das Clínicas da Faculdade de Medicina, São Paulo University, São Paulo, Brazil

**Keywords:** Echocardiography, Critical care, Ventricular function, Training course, Medical education, Children

## Abstract

**Background:**

Focused echocardiographic examinations performed by intensivists and emergency room physicians can be a valuable tool for diagnosing and managing the hemodynamic status of critically ill children. The aim of this study was to evaluate the learning curve achieved using a theoretical and practical training program designed to enable pediatric intensivists and emergency physicians to conduct targeted echocardiograms.

**Methods:**

Theoretical and practical training sessions were conducted with 16 pediatric intensivist/emergency room physicians. The program included qualitative analyses of the left ventricular (LV) and right ventricular (RV) functions, evaluation of pericardial effusion/cardiac tamponade and valvular regurgitation and measurements of the distensibility index of the inferior vena cava (dIVC), ejection fraction (EF) and cardiac index (CI). The practical training sessions were conducted in the intensive care unit; each student performed 24 echocardiograms. The students in training were evaluated in a practical manner, and the results were compared with the corresponding examinations performed by experienced echocardiographers. The evaluations occurred after 8, 16 and 24 practical examinations.

**Results:**

The concordance rates between the students and echocardiographers in the subjective analysis of the LV function were 81.3% at the first evaluation, 96.9% at the second evaluation and 100% at the third evaluation (p < 0.001). For the dIVC, we observed a concordance of 46.7% at the first evaluation, 90.3% at the second evaluation and 87.5% at the third evaluation (p = 0.004). The means of the differences between the students’ and echocardiographers’ measurements of the EF and CI were 7% and 0.56 L/min/m^2^, respectively, after the third stage of training.

**Conclusions:**

The proposed training was demonstrated to be sufficient for enabling pediatric physicians to analyze subjective LV function and to measure dIVC, EF and CI. This training course should facilitate the design of other echocardiography training courses that could be implemented in medical residency programs to improve these physicians’ technical skills and the care of critically ill patients.

## Background

Conditions that lead to hemodynamic instability occur frequently in intensive care units (ICUs) and emergency rooms (ERs) [1,2]. The evaluation of a patient’s hemodynamic status should be based on indicators that assess cardiac function and volume status, not only on physical examination findings that might be inaccurate and insufficient. Thus, using hemodynamic monitoring methods is essential, particularly the noninvasive options [3,4]. Transthoracic echocardiogram, which is a widely used method for assessing cardiac function in the ICU and ER, is a valuable tool for diagnosing, monitoring and managing critically ill patients [5,6]. Previous studies have demonstrated that the data obtained from echocardiograms, when performed by experienced echocardiographers in the ICU, may result in improved treatment in 40% of patients [7,8]. A recent review of hemodynamic monitoring in pediatric patients emphasized the importance of echocardiography as a tool for assessing cardiac function in critically ill children [9].

The concepts of critical care echocardiography (CCE) [10] and focused cardiac ultrasound (FOCUS) [11] have been developed over the last decade and consist of an examination that is performed and interpreted by the non-echocardiographer physician as an extension of the physical examination and as part of a hemodynamic monitoring assessment. The CCE comprises two levels (basic and advanced). Basic CCE, which is similar to FOCUS, is defined as an evaluation performed in a targeted and objective manner to assess a limited number of clinical issues, such as the presence of hypovolemia, left ventricular (LV) and right ventricular (RV) dysfunction, pericardial effusion (PE)/cardiac tamponade and significant valvular regurgitation [10,11].

In 2011, intensive care experts concluded that the basic CCE should be a required component of the training of every ICU physician and that a theoretical program must have a minimum of 10 hours [12]. Most recently, the American Society of Echocardiography reaffirmed the role of FOCUS as a core curriculum for all medical resident training [13]. Multiple subspecialist groups have expressed interest in using focused cardiac ultrasound, including neonatologists, pediatric/medical/surgical intensivists, anesthesiologists and trauma surgeons [12-18]. However, no consensus exists regarding a practical curriculum design for transmitting the knowledge and technical skills required to enable these physicians to perform the basic CCE/FOCUS module.

The present study was designed to test the hypothesis that a combined course of theoretical and practical training conducted under specialist supervision would enable pediatric intensivists and emergency physicians to perform targeted cardiac ultrasounds on pediatric patients, to delineate the learning curve and to determine the minimum number of practical examinations that is required for this training and thus guide the implementation of focused echocardiography training in medical residency programs.

## Methods

### Selection of participants

The patients were considered to be eligible for the study if they were between one month and 14 years of age. Preference was given to the inclusion of patients with hospitalizations related to hemodynamic instability; however, in the absence of these patients, we included patients with other causes of hospitalization, except children with a congenital heart defect. The project was approved by the ethics committee of the institution, and free and informed consent was obtained from all of the patients’ legal guardians before each participant was included in the study.

The number of tests required for the study to achieve 95% confidence and 80% power was calculated using the variable cardiac index (for its clinical utility and technical difficulty); 16 tests in each stage of the evaluation were shown to be necessary. Among the 20 ICU/ER physicians who had volunteered to participate (13 intensivists and seven emergency physicians), 16 were randomly selected to attend the training (10 intensivists and six emergency physicians). Among the 16 participating physicians, the median age was 33 years (28-50 years), two were male and 14 were female, and they included both recent graduates and experienced physicians. All of the participants had at least 1 year of work experience in a pediatric ER or ICU. The pediatric physicians in training did not have prior ultrasound and echocardiography experience.

### Course model and curricular content

**Figure 1:**
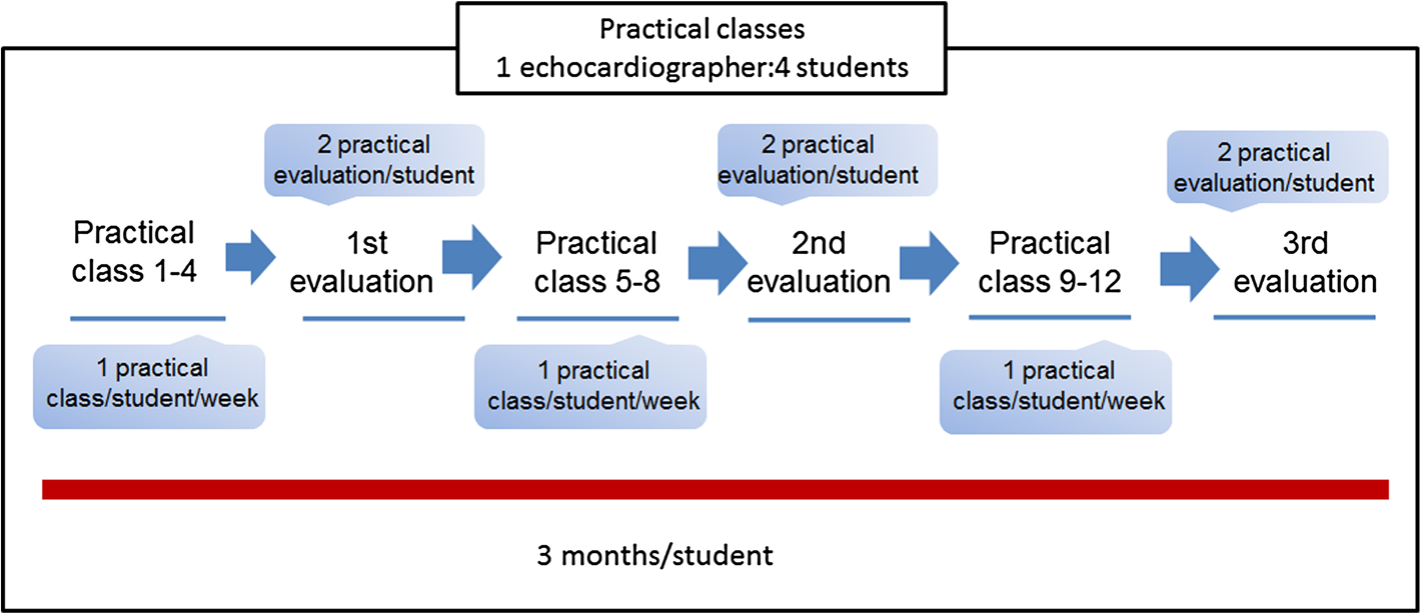
**Schedule of the practical lessons and evaluation plan.** Two similar modules (1 and 2).

The training curriculum, consisting of 10 theoretical lessons followed by 12 practical sessions, was conducted in two similar groups (eight physicians/group), and each section lasted for three months. Each student was submitted to one practical class/week and remained in training for 3 months (Figure 1). The training was conducted and supervised by two advanced pediatric echocardiographers, according to the American Society of Echocardiography guidelines [19].

The theoretical lessons consisted of 10 hours of training (one hour per lesson) that comprised expositive classes and discussions of videos and images; these lessons addressed the entire content to be evaluated in the practical training. The curriculum was based on the main topics determined by previous experts’ consensus [10] and included volemia evaluation by measuring the dIVC, qualitative analyses of the LV and RV functions, evaluation of PE/cardiac tamponade and valvular regurgitation and quantitative analysis of the LV function by measuring the ejection fraction (EF) and cardiac index (CI) (Table 1).

**Table 1:** Theoretical content of the training

	
Basic principles of ultrasonography/echocardiography	
Handling of portable echocardiogram equipment	
Essential cardiac anatomy	
Obtaining echocardiographic images	
Pulsed Doppler and color flow mapping	
Subjective assessment of left ventricular systolic function and evaluation of the shortening/ejection fraction	
Subjective assessment of the right ventricle systolic function	
Volume status evaluation-distensibility index of the inferior vena cava (dIVC). [dIVC = Dmáx – Dmin/Dmin]	
Assessment of cardiac output-aortic velocity-time integral ratio and left ventricle outflow tract diameter	
Evaluation of the cardiac valves and the pericardium	

The practical lessons were divided into subgroups attended by four students who were supervised by one echocardiographer, for a total of 12 lessons per student (one lesson/week), with each lesson lasting four hours. We opted to perform only one practical class/week/student to allow a higher patient turnover. The students in training conducted two full targeted echocardiographic examinations of different patients in each of the 12 practical lessons; thus, at the end of the training period, each student had performed 24 examinations under supervision (Figure 1). Each student also participated in the real-time assessment of the examinations conducted by the three other students in their subgroup, totaling 96 examinations that were interpreted by each student at the end of the course. The physicians in training did not perform any echocardiographic examinations other than the practical and evaluation sessions.

All of the practical classes were conducted in the pediatric ICU that was classified as Level I by the Society of Critical Care Medicine [20]. The facility has an 18-bed ICU, with an average of 40 inpatients/month, providing care to medical and surgical patients (except for patients with congenital heart defects, who are treated in another specified unit). Mechanical ventilation was used in nearly 60% of the patients, with vasoactive drug administration greater than 30% and a mortality rate of approximately 10%.

The portable echocardiogram equipment employed throughout the entire training program (Sonosite® MicroMaxx model, Sonosite, Inc., Bothell, Washington, USA) allowed measurements and calculations using M-mode, two-dimensional and Doppler techniques. The practical training sessions and evaluations were performed using image acquisition through the parasternal (long and short axis), subcostal (four-chamber and inferior vena cava) and apical (four- and five-chamber) windows. The content of the training is presented in Table 2.

**Table 2:** Practical content of the training

	
**By M**-**mode:**	
Left ventricle systolic and diastolic diameter (parasternal long axis)	
Left ventricle shortening fraction (parasternal long axis)	
Left ventricle ejection fraction (parasternal long axis)	
Distensibility index of the inferior vena cava (subcostal view)	
**By 2 - dimensional imaging:**	
Qualitative assessment of left ventricular function (normal, mild, moderate or severe depressed)	
Qualitative assessment of right ventricular function (normal, mild, moderate or severe depressed)	
Presence and severity of pericardial effusion (absent, small, moderate or severe)	
Left ventricle outflow tract diameter (parasternal long axis)	
**By Doppler:**	
Velocity-time integral of aortic flow (apical 5-chamber view)	
**By color flow mapping:**	
Presence and severity of mitral and tricuspid valve regurgitation (absent, mild, moderate or severe) (apical 4-chamber view)	

### Echocardiography variables

**Figure 2:**
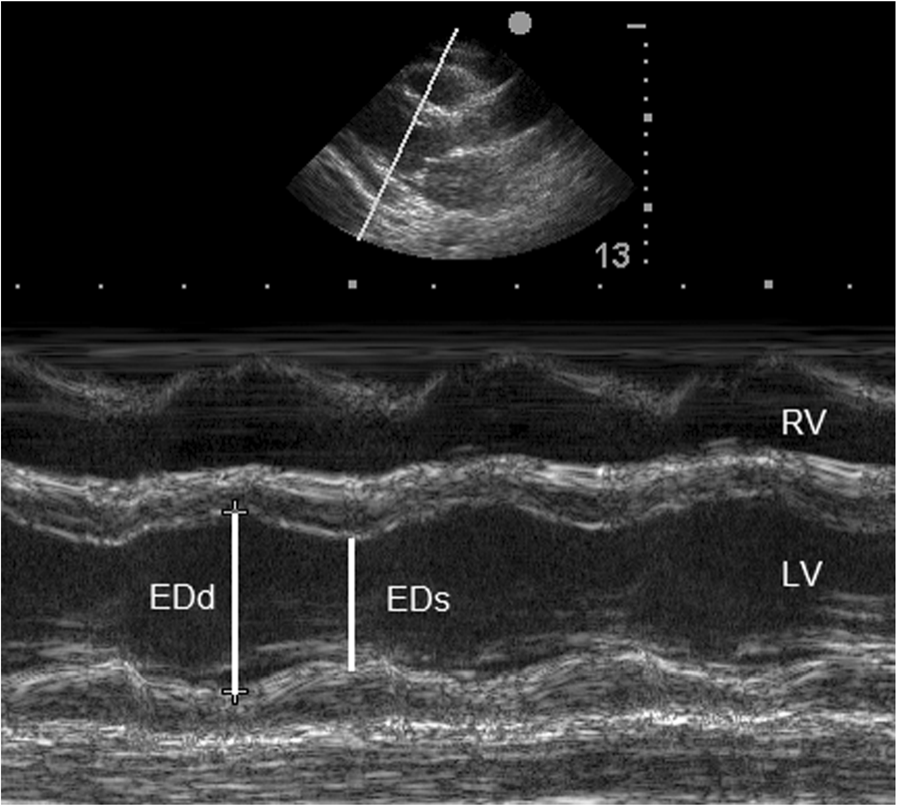
**Measurement of the ejection fraction using M-mode in the parasternal long-axis view.** The LV end-diastolic internal dimensional (EDd) is measured at the largest dimension, and the LV end-systolic internal dimensional (ESd) is measured at the smallest dimension. RV, right ventricle.

The LV function, assessed both qualitatively and quantitatively, was graded and classified subjectively through visual evaluation using two-dimensional images as follows: 0 - normal (EF greater than 55%), 1 - slight dysfunction (EF between 40% and 55%), 2 - moderate dysfunction (EF between 30% and 40%) and 3 - severe dysfunction (EF less than 30%). To calculate the EF, end-systolic and end-diastolic LV internal diameters were measured by M-mode using the inner edge technique at the level of the mitral leaflet tips in the parasternal long-axis view (Figure 2). The RV function was assessed only qualitatively and graded subjectively by two-dimensional mode using the same classification described for LV function.

The dIVC was calculated based on the respiratory changes in the IVC diameter using the following formula: dIVC = (maximum diameter IVC – minimum diameter IVC)/minimum diameter. This index was indicative of individual volume responsiveness when greater than 18% and of volume unresponsiveness when less than 18% [21].

**Figure 3:**
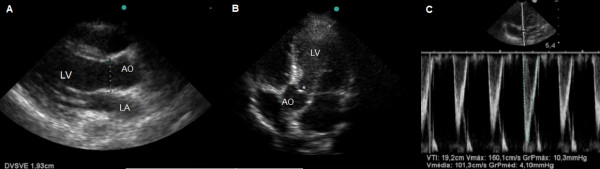
**Cardiac index calculation. A**- Measurement of the left ventricle outflow tract by the parasternal long-axis view (diameter of the aortic annulus during systole). **B and C**- Measurements of aortic VTI through pulsed wave sample volume (*) at the aortic annulus (apical 5-chamber view). Ao, aorta; LA, left atrium; LV, left ventricle.

To calculate the CI, the aortic velocity-time integral (VTI) ratio (apical five-chamber view) and the LV outflow tract (LVOT) diameter (parasternal long-axis view) were measured (Figure 3).

**Figure 4:**
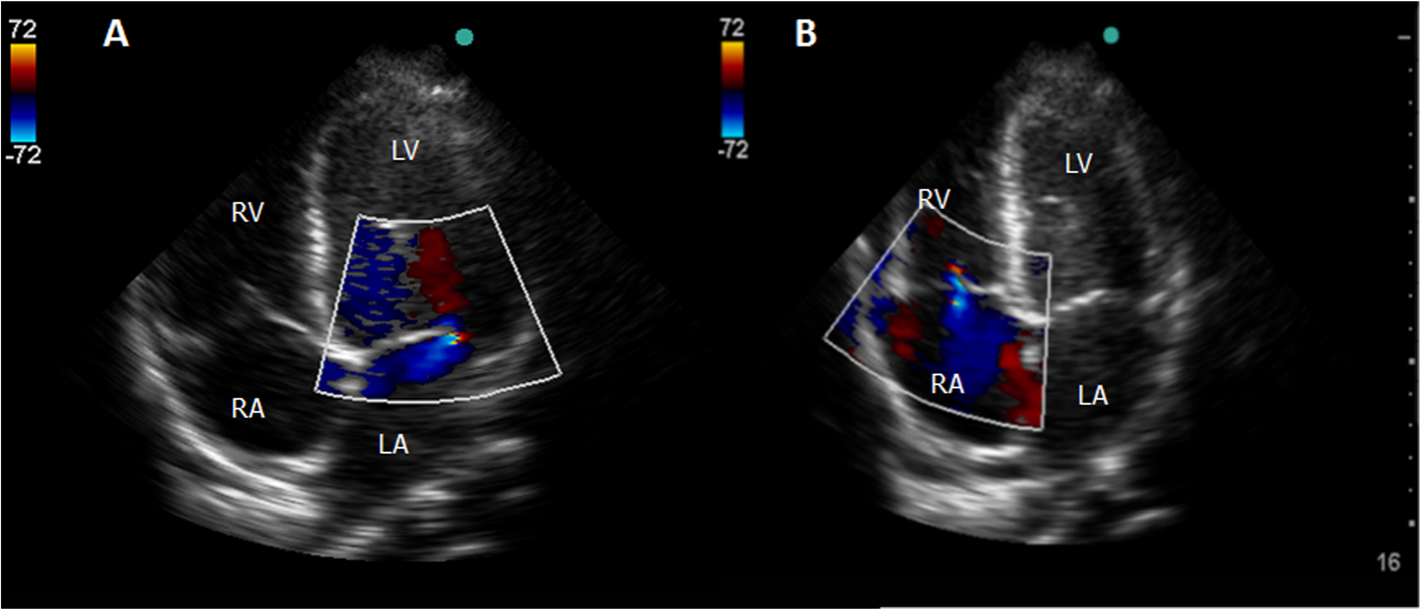
**Valvular regurgitation. A**- Mild MR by color Doppler (apical 4-chamber view). **B**- Moderate TR by color Doppler (apical 4-chamber view). LA, left atrium; LV, left ventricle; RA, right atrium; RV, right ventricle.

The tricuspid regurgitation (TR) and mitral regurgitation (MR) were evaluated using color flow Doppler and were graded qualitatively as absent, mild (when the jet reached less than one-third of the atrial cavity), moderate (when the jet reached two-thirds of the atrial cavity) and severe (when the jet reached the atrial roof) (Figure 4).

Pericardial effusion was classified based on the distance between the heart and parietal pericardium as absent, mild (<0.5 cm), moderate (0.5-2 cm) and important (>2 cm).

### Assessment

The evaluation of the students was practical and consisted of the completion of a targeted echocardiographic examination with subjective and objective analyses. The evaluation was documented via image recording and by completing a form listing the variables in question (Table 3). The students and the echocardiographer performed the examination measurements less than an hour apart from each other; both the student and the echocardiographer were blinded to the other’s results.

**Table 3:** Evaluation form - data obtained and echocardiographic views

**Variable-Echo**		**Window**		
LV diastolic diameter (cm)		Parasternal long axis		
LV systolic diameter (cm)		Parasternal long axis		
Shortening fraction (%)		Parasternal long axis		
Ejection fraction (%)		Parasternal long axis		
Largest diameter of the inferior vena cava (cm)		Subcostal		
Smallest diameter of the inferior vena cava (cm)		Subcostal		
Distensibility index of the IVC (dIVC)*		Subcostal		
Left ventricular function (0 3)		Parasternal/subcostal/apical		
Right ventricular function (0 3)		Parasternal/subcostal/apical		
Pericardial effusion (0 3)		Parasternal/subcostal/apical		
Aortic velocity time integral (VTI) (cm)		Apical 5-chamber		
Left ventricle outflow tract diameter (cm)		Parasternal long axis		
Cardiac index (L/min/m2)		Apical/Parasternal		
Stroke volume (mL)		Apical/Parasternal		
Mitral valve regurgitation (0 3)		Apical 4-chamber		
Tricuspid valve regurgitation		(0 3) Apical 4-chamber		
* <18% or >18%	0 – normal/absent	1 – mild	2 – moderate	3 – severe
**PE		**TR/MR		
Mild: <0,5 cm		Mild: <1/3 atrial cavity		
Moderate: 0,5 2 cm		Moderate: 1/3 2/3 atrial cavity		
Severe: >2 cm		Severe: reaches atrial roof		

**Graduation criteria for pericardial effusion (PE), mitral regurgitation (MR) and tricuspid regurgitation (TR).

Each student was tested at three different intervals throughout the training course: at the end of the eighth training examination (first evaluation), after the 16th examination (second evaluation) and after the 24th examination (third evaluation). For each evaluation, the tested student performed echocardiographic examinations of two different patients. The time allotted to complete each examination was 10 minutes. Figure 1 illustrates the training and evaluation sequence.

### Statistical analysis

Statistical analysis of the continuous variables (EF and CI) was performed by comparing the average absolute difference between the evaluations by each student and the evaluations by the echocardiographer using repeated-measures analysis of variance. For the other measures (dIVC, subjective analyses of RV and LV functions, PE and valvular regurgitation), categories of concordance or nonconcordance with the cardiologist were created (Table 3). These differences were compared before and after the training (i.e., the first and final evaluations) using the generalized estimation equation with a binomial distribution and a logit liaison function; for the models that yielded statistical significance, the analysis was supplemented by Bonferroni multiple comparisons to identify which evaluations (1st, 2nd and 3rd) displayed differences. Significance was set at the 5% level.

## Results

**Figure 5:**
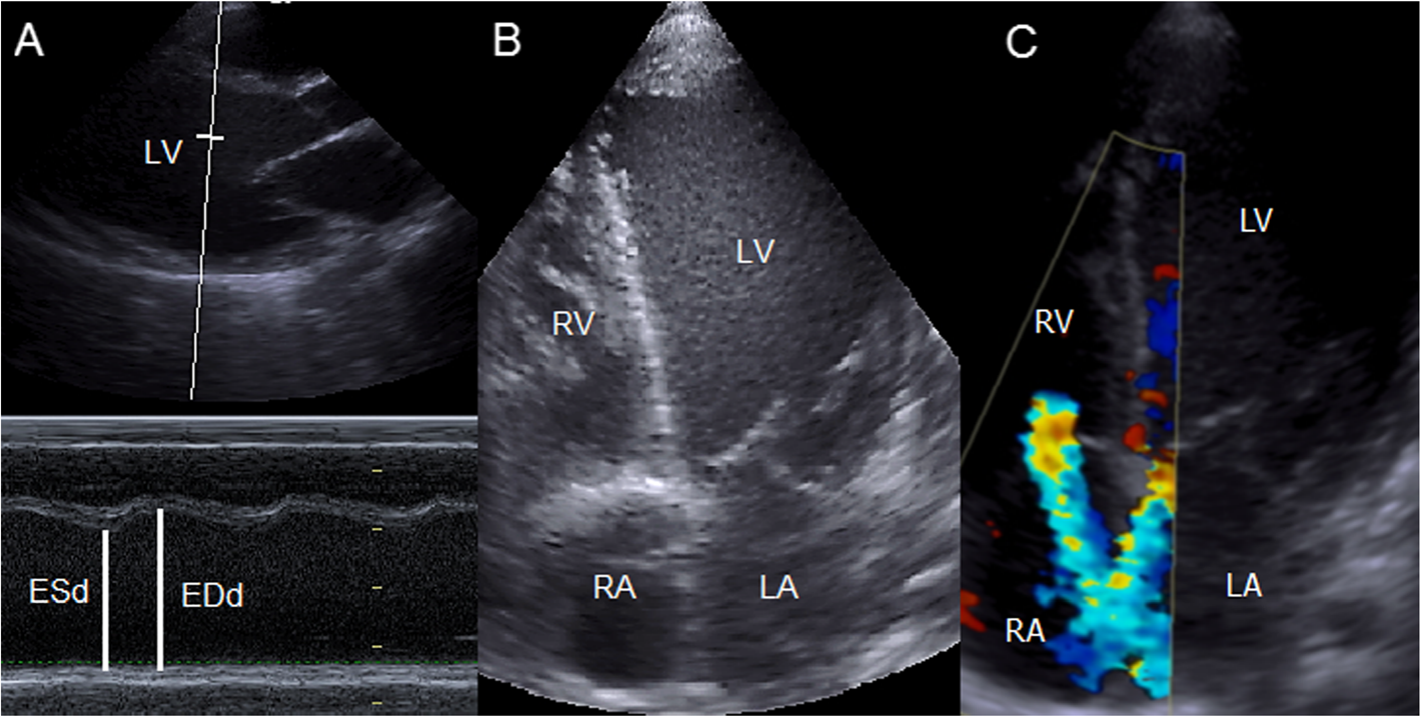
**Four-year-old patient with important LV dysfunction. A**- EF of 21% by M-mode parasternal long-axis view. **B**- Significant LV dilation and **C**- moderate TR seen from the apical 4-chamber view. EDd, end-diastolic diameter; ESd, end-systolic diameter; LA, left atrium; LV, left ventricle; RA, right atrium; RV, right ventricle.

We conducted 96 practical examinations during the evaluations. Among the patients, 44% were male, and 56% were female, with a median age of 63 months. A total of 66 (69%) patients underwent mechanical ventilation, and 44 (45%) received vasoactive agents. The most frequent clinical diagnosis was septic shock (39%), followed by acute respiratory failure (33%) and neurological disease (16%). There was one case of pulmonary hypertension. We observed TR in 35 (37%) subjects, with 27 (28%) classified as mild, 5 (5%) as moderate (Figure 5) and 3 (3%) as severe. MR was found in 21 (22%) subjects, with all classified as mild. PE occurred in 15 (16%) subjects, LV dysfunction in 10 (10%) (Figure 5), and RV dysfunction in only two (2%). There were no cases of cardiac tamponade.

At the first evaluation, the students’ inability to perform echocardiographic measurements because of the technical difficulty of image capture was 15.6% for the EF and CI, with great improvement throughout the training, as demonstrated by reductions in the inability rate to 6.3% and 3.1%, respectively, in the second and the third stages of evaluation. The same phenomena were observed for dIVC, MR and TR.

The concordance rates between the students and echocardiographers in the subjective analysis of LV function were 81.3% at the first evaluation, 96.9% at the second evaluation and 100% at the third evaluation (p < 0.001 for the 1st evaluation vs. the 3rd evaluation). Regarding the subjective analysis of LV function, when we comparatively assessed the first and second evaluations and the second and third evaluations separately, we observed no significant differences (p = 0.1 and 0.9, respectively). For the RV subjective function evaluation, we observed concordance rates of 93.8%, 100% and 100% at each of the steps, respectively. This analysis was affected by the limited number of subjects (two cases) who had RV dysfunction (Table 4).

**Table 4:** Concordance between students and echocardiographers in the three-phase evaluations

		**Evaluation**						
**Variable**	**Concordance**	**1**		**2**		**3**		**p**
		**n**	**%**	**n**	**%**	**n**	**%**	
dIVC	Disagree	8	53,3	3	9,7	4	12,5	0,004
	Agree	7	46,7	28	90,3	28	87,5	
	Total	15	100	31	100	32	100	
LVF	Disagree*	6	18,8	1	3,1	0	0	<0,001
	Agree	26	81,3	31	96,9	32	100	
	Total	32	100	32	100	32	100	
RVF	Disagree*	2	6,3	0	0	0	0	#
	Agree	30	93,8	32	100	32	100	
	Total	32	100	32	100	32	100	
PE	Disagree*	2	6,3	0	0	5	15,6	0,258
	Agree	30	93,8	32	100	27	84,4	
	Total	32	100	32	100	32	100	
MR	Disagree*	9	29	2	6,7	3	9,4	0,043
	Agree	22	71	28	93,3	29	90,6	
	Total	31	100	30	100	32	100	
TR	Disagree*	8	25,8	7	22,6	6	18,8	0,758
	Agree	23	74,2	24	77,4	26	81,3	
	Total	31	100	31	100	32	100	

dIVC, distensibility index inferior vena cava; LVF, subjective left ventricular function; MR, mitral regurgitation; PE, pericardial effusion; RVF, right ventricular function; TR, tricuspid regurgitation.

*The non-concordance was one degree for these variables in the three assessment phases.

The means of the absolute differences between the students’ and echocardiographers’ EF measurements were 9.1% at the first evaluation, 8% at the second evaluation and 7% at the third evaluation (p = 0.6), and the medians were 8.8%, 6% and 5.2%, respectively.

For the dIVC measurement, we observed a concordance of 46.7% at the first evaluation, 90.3% at the second evaluation and 87.5% at the third evaluation (p = 0.004). Comparing the first and second evaluations, there was a significant difference (p = 0.006), which was not found when the second and third evaluations were compared.

The means of the differences in the CI measurements were 1.31 L/min/m^2^ at the first evaluation, 1.29 L/min/m^2^ at the second evaluation and 0.56 L/min/m^2^ at the third evaluation (p = 0.001), and the medians were 1.1 L/min/m^2^, 0.9 L/min/m^2^ and 0.5 L/min/m^2^, respectively. The statistical analysis revealed a significant reduction in the difference in the mean CI measured by the students compared with the mean CI measured by the echocardiographers only in the final training phase (1st vs. 3rd evaluations).

The MR identification, with the correct degree of classification (mild, moderate or severe), between the students and echocardiographers were 71%, 93.3% and 90.6%, respectively, during the three evaluations (p = 0.04 between the 1st and 3rd evaluations; p = 0.05 between the 1st and 2nd evaluations). The TR identification and classification were correct in 74%, 77% and 81% of the cases, respectively (p = 0.75).

**Figure 6:**
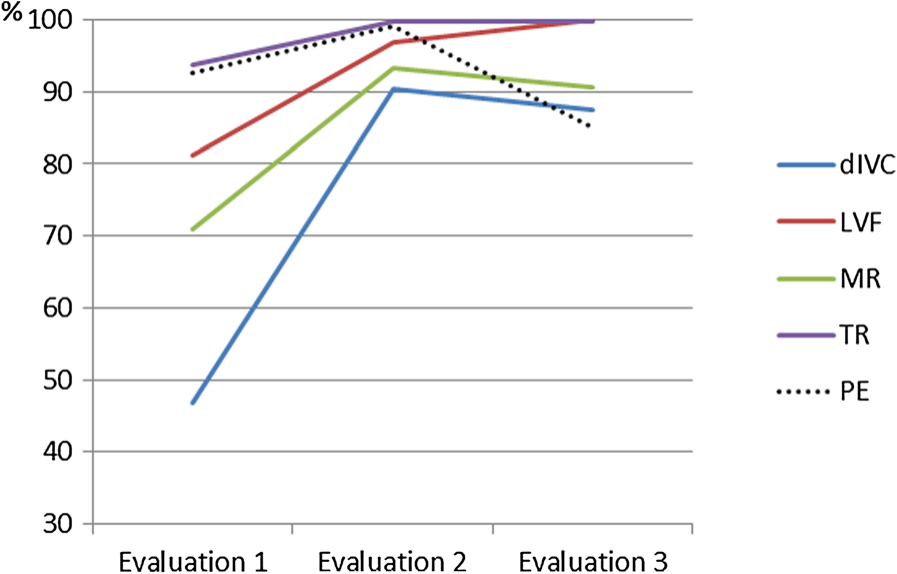
**Concordance evolution analysis through the training.** dIVC, distensibility index of inferior vena cava; LVF, left ventricular function; MR, mitral regurgitation; PE, pericardial effusion; TR: tricuspid regurgitation.

The PE evaluation displayed improved concordance between the students and echocardiographers when the first and second evaluations were compared (94% vs. 100%), with a drop in concordance to 84% at the third evaluation (p = 0.25). There were no cases of severe PE or cardiac tamponade in any phase of the study. Figure 6 shows the evolutionary analysis of the concordances between the students and echocardiographers for these variables during the training.

After completing the training curriculum, the students completed a questionnaire in which they were asked whether they felt capable of performing the various assessments. Of the 16 students trained, all felt capable of performing subjective and objective analyses of LV function, measurements of the dIVC index and identification of PE. Fourteen (87%) students considered themselves capable of assessing RV function, and 12 (75%) considered themselves capable of assessing valvular regurgitation.

## Discussion

The evaluation of LV function is an important assessment for the noncardiologist physician, with great clinical utility and can be performed subjectively without any specific measurements. In the present study, we observed increasingly high rates of concordance between the LV subjective evaluations performed by students and echocardiographers throughout the training session, with a high concordance rate (96.9%) even at the second stage of the evaluation. Similarly, Melamed et al. reported that intensivists were able to distinguish normal LV function from altered function in 86% of their adult patients [16]. In addition, Vignon et al. demonstrated a correct assessment of the LV function in 92% of the patients [22], and Manasia et al. showed accurate determinations in 84% of the patients [17]. Studying pediatric patients, Spurney et al. assessed the concordance of subjective LV function performed by pediatricians and cardiologists and demonstrated a strong correlation (96%) [23]. Longjohn et al. also demonstrated a good interobserver agreement (κ = 0.87) between pediatricians and echocardiographers in subjectively differentiating normal LV function from reduced LV function [24].

The clinical utility of EF measurements for the management of critically ill patients has been established in previous pediatric studies [25,26]. We obtained a strong correlation between the quantitative EF measurements performed by the students and the echocardiographers in all three phases of training, with a mean of the absolute difference that was consistently less than ten EF points. Similar results were also reported by Pershad et al. in a pediatric population, in which the mean difference in the shortening fraction of the LV measurements was 4.4% after a brief theoretical/practical training course [27].

Regarding the subjective analysis of RV function, we observed that the concordance rate between students and echocardiographers was high in the 3 stages of evaluation; unfortunately, in our sample, there were only 2 children with RV dysfunction. Among the students, only 14 (87.5%) felt they were capable of performing this assessment; therefore, our data do not allow us to make conclusions about the empowerment and learning curve of students on the subjective analysis of RV function.

The measurement of CI is widely accepted and used as a hemodynamic monitoring tool in ICUs [28]. Minimally invasive devices, which are based primarily on pulse pressure analyses, are being developed for the measurement of CI in the adult patient population [29-31]. However, due to technical difficulties related to the unique physiological characteristics of children, these methods do not present an effective option in the arsenal of hemodynamic monitoring devices for the pediatric population. Although the calculation of a CI value via a transthoracic echocardiogram requires measurements of the LVOT diameter and the aortic VTI and is therefore considered to be technically challenging, we obtained a good correlation after the third evaluation with a mean difference of only 0.56 L/min/m^2^ between the measurements performed by the students and the echocardiographers. Evaluation of the CI by trained physicians in our study was performed in an innovative manner and demonstrated relevant results. This assessment might provide a new option in the arsenal for the hemodynamic monitoring of critically ill children, which emphasizes the need for extensive training that we observed in our study.

Respiratory changes in the diameter of the IVC in patients on mechanical ventilation are related to the individual’s volemic state; hypovolemic patients present greater effects of positive pressure ventilation in venous return and a greater variation in the diameter of the IVC [21,32]. In our study, we observed a strong correlation at the second evaluation (16 training examinations), with a 90% concordance in the patient classification as volume-responsive (dIVC greater than 18%) or volume-unresponsive (dIVC less than 18%). In a pediatric study, Pershad et al. reported a strong correlation between the IVC diameter measurements performed by students in training compared with the measurements obtained by experienced echocardiographers; however, neither the dIVC nor another dynamic measurement of the IVC was evaluated during this study [27].

With respect to the evaluation of PE, we observed a decrease in concordance at the third evaluation. This reduced concordance rate could be explained by the small number of PEs that we had at this evaluation phase. Three cases were classified as mild by the echocardiographers but regarded by the students as absent. This misclassification could be attributed to the difficulties in distinguishing mild effusion from epicardial fat; most likely this type of PE would not have yielded any clinical implications for patient management. The literature reveals data that indicate that non-echocardiographer physicians can identify moderate and severe PE and cases of cardiac tamponade [22,33]. We believe that PE diagnosis could be performed by the ICU physician, especially in cases of large volumes; however, the limited number of cases, together with the absence of severe PE and cardiac tamponade, does not allow us to confirm this statement.

The analysis of valve regurgitation does not appear to be of great clinical importance in the emergency evaluation of critically ill children because of the rare occurrence of serious regurgitation with hemodynamic repercussions in children who do not present congenital heart disease. However, we performed the evaluation of MR and TR with the primary objective of addressing the student’s ability to identify TR, for a further measurement of the pulmonary pressure through the TR. We observed a good concordance rate in identifying MR after 16 training examinations; however, we did not see major improvements in the identification of TR during the training. Another relevant observation is that only 75% of students felt capable of detecting and grading valve regurgitation after the training. Therefore, although our work is the first to report the identification and graduation of MR and TR by pediatricians, our data do not allow us to state that this training was accomplished in a successful manner, and further analysis in this direction is required.

Royse et al. trained 100 students using multimedia presentations and practical lessons in healthy volunteers, without training the students on actual patients [34]. The students’ assessment was performed by analyzing videos and yielded concordance rates of 95% for the evaluation of volemia and 99% for the analysis of LV function. Recently, Tanzola et al. reported on the training of 10 anesthesiology residents via theoretical sessions and “hands-on” sessions using normal subjects. The training included subjective analyses of RV and LV function, volume assessment and pericardial disease, and the students’ evaluations were performed using 50 multiple-choice questions and videos. The results were presented as positive by improving post-test scores; however, there was no practical evaluation of the training [35]. The examination of ICU or ER patients in loco presents technical difficulties related to the capture of echocardiographic images due to factors specifically related to critically ill patients, such as the use of mechanical ventilation, limited mobility and difficulty in positioning the patient for the examination; these issues were not considered in the above-mentioned studies.

The present study, compared with previous studies, is noteworthy because it was conducted in the ICU and the examinations were performed on critically ill patients and provided practical training to a greater number of professionals. Our study is the first to establish the learning curve throughout the training of the pediatricians. The present study demonstrates that pediatric intensivists and emergency physicians are capable of performing the focused bedside echocardiography approach in critically ill children and emphasizes that whenever doubt exists regarding the presence of anatomical or functional abnormalities, the case should always be discussed with and reassessed by a pediatric echocardiographer [13,14].

### Limitations

The primary limitations of the present study are as follows: 1) the echocardiography examinations were performed on selected patients based on a predefined training schedule; thus, the study was conducted without including all of the echocardiographic possible abnormalities (e.g., there were no cases of cardiac tamponade or severe PE) during the training and evaluation stages, which limited our assessment of the learning curve regarding these echocardiographic alterations; 2) the design and sampling protocol of the study did not facilitate the evaluation of certain factors, such as accuracy and precision.

## Conclusions

This study suggests that the proposed theoretical-practical training curriculum conducted under the supervision of an experienced echocardiographer is sufficient to enable pediatric intensivists and emergency physicians to perform the primary evaluations suggested as critical care echocardiography/FOCUS and to also objectively measure LV function through the evaluation of ejection fraction and cardiac index.

The analysis of the learning curve demonstrated that 16 supervised practical examinations were effective in instructing the physicians to analyze both qualitative and quantitative function of the LV through EF and to also calculate the dIVC. With respect to the CI measurement, more extensive training was required, involving the performance of 24 practical examinations.

The proposed training can serve as a basis for supporting the inclusion of basic echocardiography training programs for physicians of several medical specialties during medical residency or even in separate courses to improve these physicians’ technical skills and, therefore, the care they provide to critically ill patients.

## Abbreviations

CCE: Critical care echocardiography; CI: Cardiac index; CO: Cardiac output; dIVC: Distensibility index of the inferior vena cava; EF: Ejection fraction; ER: Emergency room; FOCUS: Focused cardiac ultrasound; ICU: Intensive care unit; LV: Left ventricle; LVF: Subjective left ventricular function; LVOT: Left ventricle outflow tract; MR: Mitral regurgitation; PE: Pericardial effusion; RV: Pight ventricle; RVF: Subjective right ventricular function; VTI: Velocity-time integral; TR: Tricuspid regurgitation.

## Competing interests

The authors declare that they have no competing interests.

## Authors’ contributions

HG and AD were responsible for the design of the study as well as actively participated in all phases of execution and manuscript writing. SM and AL were the two echocardiographers who performed the theoretical-practical training for the students and also drafted the manuscript. WC, JA and CS are the chiefs of the intensive care unit, radiology unit and emergency room unit, respectively, and supported on the design and coordination of the study and also helped to draft the manuscript. RP participated in the design of the study and performed the statistical analysis. All authors read and approved the final manuscript.

## Pre-publication history

The pre-publication history for this paper can be accessed here:

http://www.biomedcentral.com/1472-6920/14/25/prepub
